# DNA- and RNA-based bacterial communities and geochemical zonation under changing sediment porewater dynamics on the Aldabra Atoll

**DOI:** 10.1038/s41598-022-07980-0

**Published:** 2022-03-11

**Authors:** Avril Jean Elisabeth von Hoyningen-Huene, Dominik Schneider, Dario Fussmann, Andreas Reimer, Gernot Arp, Rolf Daniel

**Affiliations:** 1 Genomic and Applied Microbiology and Göttingen Genomics Laboratory, Institute of Microbiology and Genetics, Georg-August University of Göttingen, Grisebachstr. 8, 37077 Göttingen, Germany; 2 Geobiology, Geoscience Centre, Georg-August University of Göttingen, Göttingen, Germany

**Keywords:** Microbiology, Microbial communities, Environmental microbiology, Biodiversity, Microbial ecology

## Abstract

The remote Aldabra Atoll, Seychelles, provides the rare opportunity to study bacterial communities in pristine carbonate sediments across an entire biome. The four sampled sites cover sand with high porewater exchange, bioturbated silt and mud with intermediate exchange, as well as a seasonally and episodically desiccated landlocked pool. As sediments harbour dead cells and environmental DNA alongside live cells, we used bacterial 16S rRNA gene and transcript analysis to distinguish between past and present inhabitants. Previously described laminated sediments mirroring past conditions in the Cerin, France could not be retrieved. Thus, the aim was adjusted to determine whether bacterial community composition and diversity follow typical geochemical zonation patterns at different locations of the atoll. Our data confirm previous observations that diversity decreases with depth. In the lagoon, the bacterial community composition changed from *Pseudomonas* dominating in the sand to diverse mixed surface and sulphate reduction zones in the anaerobic mud with strongly negative Eh. The latter correlated with high total alkalinity, ammonia, and total sulphide, alongside a decrease in SO_4_^2−^/Cl^−^ and high relative abundances of sulphate reducing (*Halo-) Desulfovibrio*, sulphur oxidizing *Arcobacteraceae*, photo(hetero)troph *Cyanobacteria*, *Alphaproteobacteria*, and fermenting *Propionigenium*. In contrast to expectations, deeper mud and pool sediments harboured high abundances of *Halomonas* or *Alphaproteobacteria* alongside high C/N and increased salinity. We believe that this atypical community shift may be driven by a change in the complexity of available organic matter.

## Introduction

Microbial communities are major drivers of the global biogeochemical cycles. These cycles are based on a limited set of elements, which are used in the energy transduction pathways, depending on their abundance and the availability of oxygen^1^. In sediments, this results in a succession of redox zones. Once oxygen is depleted, the electron acceptors change to nitrate, followed by manganese, iron and sulphate. In the deepest sediments, CO_2_ is ultimately reduced to methane^2,3^. Changes in microbial community composition are favoured according to this standard biogeochemical zonation. Due to fluctuations and advection of porewaters and corresponding changes in ion concentrations, as well as bioturbation and bio-irrigation, they may not necessarily appear as clearly separated zones^4,5^. This leads to a highly dynamic sediment environment with diverse bacterial communities in micro-niches characterized for instance by different levels of oxygenation^5^. The most applied approach to studying total bacterial community assemblies is the use of the 16S rRNA gene as taxonomic marker. The DNA-based approach has been shown to yield overlapping signals of past and present microbial communities, depending on organic matter degradation rates in the sediment. This means that in addition to DNA of present microbes, signals from extracellular DNA, dormant or dead microbes may be detected^6^. Biases in the abundance data may also derive from differences in 16S rRNA gene copy numbers within different bacterial genomes, however, appropriate normalization is not yet available^7^. Analysis of 16S rRNA transcripts can be utilized as an indicator for the protein synthesis potential of the bacterial community, and as such describes the potentially active part of the community^8,9^. While this method cannot distinguish between growth phase, metabolic activity, or dormancy, it does exclude signatures from dead cells and extracellular DNA (eDNA). In line with this, it has been shown that the RNA-based fraction reflects the active proportion of the community more accurately^9–11^. We therefore applied this approach to disentangle past (dead) and present (live) signatures in sediments from the Aldabra Atoll.

Marine sediments have been studied ranging from continental coastlines^12^ to deep-sea sediments^13^. Atolls represent isolated island biomes, which are rarely investigated often due to their remoteness. Studies on microbes have been undertaken i.e., at the Line Islands^14^, including the largest atoll Kiritimati^15,16^, or at Xuande Atoll^17^. They focus on microbial communities in microbial mats^16^, the water column^18^ and associated coral reefs^14^. Sediments are only covered regarding the uppermost 5–10 cm of depth^15,17^, and mostly provide only limited (or no) information on the sedimentary facies and porewaters. Geochemical gradients and bacterial community composition in tropical atoll sediments reaching below 10 cm of depth have, to our knowledge, not been assessed to date.

The Aldabra Atoll, Seychelles, is the second largest raised limestone atoll in the world. Situated approximately 420 km north of Madagascar, Aldabra covers an area of 365 km^2^ of which 155 km^2^ are attributed to the island rim^19^. The large, shallow lagoon experiences tidal fluctuations with an average amplitude of 2.74 m. In comparison to Pacific or other Indian Ocean atolls this corresponds to a 2- to fivefold higher tidal range^20^. As a consequence, sediments can accumulate in protected embayments, where they are exposed and often drained during low tide. In a previous study the sediments were described as pure lime mud with similarities to the laminated lithographic limestone of the Cerin paleoenvironment^21^. In 2012, the lagoons’ sediment types and benthic cover were determined using remote sensing and ground reference points for spatial models^22^. The Pleistocene limestone basement was described in detail by Braithwaite^23^ and the biota, including the giant tortoises^24^ and (macro-)biota of the soft sediments^25^, have received some attention. The most recent studies on microbial communities were performed 30–40 years ago. They focused on microbial mats covering the limestone within the lagoon or the landlocked pools and the rates at which they fix nitrogen in the intertidal zone^26,27^. For the identification of dominant blue-green algae, such as *Hyella balani*, *Lyngbya* sp., and *Schizothrix*, the authors relied on phenotypic description^28^.

Based on previous observations from Aldabra, this study set out to characterize the laminated limestone mud from a geochemical and microbiological perspective. These present-day data would provide a window into the past, by mirroring conditions in the Cerin. In addition, cross-sectional studies using sediment cores across an entire atoll are scarce. We therefore used this unique opportunity to capture the diversity and variability of the sediments and their native bacterial communities. With the aim to recover laminated mud cores, four sediment depth profiles with different tidal and depositional histories were assessed. All sites were sampled during the day and at low tide. We hypothesized that, firstly, the sediment geochemical profiles and associated bacterial communities should follow the classical sediment zonation. Secondly, we expected to observe a decrease in bacterial diversity with increasing sediment depth, which is common in sediments^12,13^. Thirdly, the potentially active (RNA-based) bacterial community in comparison to the total (DNA-based) bacterial community should highlight taxa, which contribute to the main geochemical processes observed in the porewater profiles. The total community provides an extended picture of the system, additionally preserving past members within the community. This is the first study covering not only surface sediments, but multiple depth profiles of the remote Aldabra Atoll using both geochemical profiling and next-generation sequencing.

## Results

### Sediment cores, porewater and bulk sediment geochemistry

**Figure 1:**
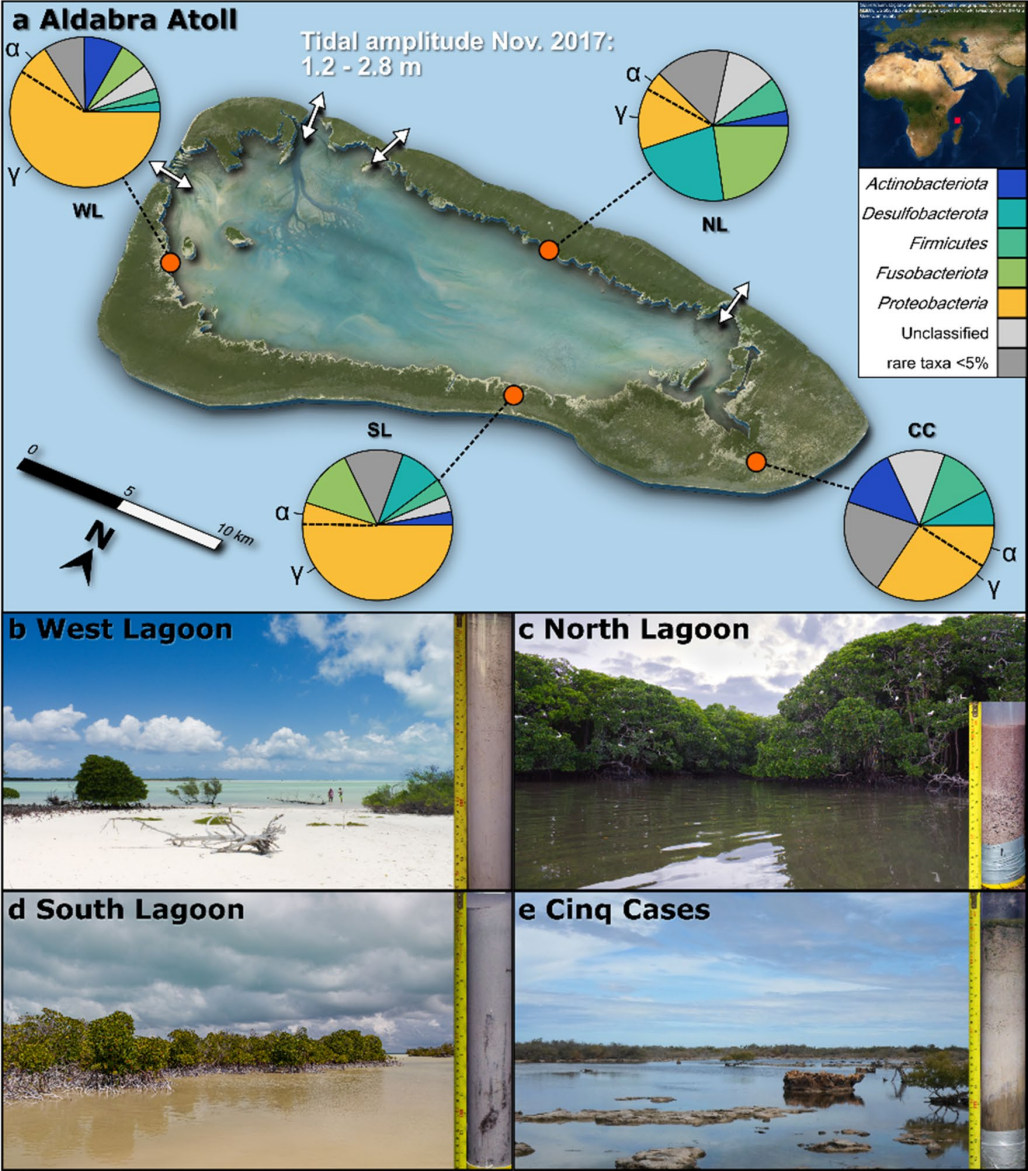
Aldabra Atoll, sampling sites and most abundant bacterial phyla (> 5%) at each site. (**a**) Map of the Aldabra Atoll showing the main tidal channels (white arrows), tidal amplitude and four sampling sites. The global location is indicated in the top right corner, on a map generated using ArcGIS Desktop and Esri World Imagery (July 17, 2020). Most abundant bacterial phyla across all samples are shown in pie charts by sampling site: All taxa below 5% relative abundance were summarized as rare taxa. (**b**–**e**) Sampling sites and a representative core for each location. (**b**) The sediment cores were sampled near the bottom left corner of the image when sands were completely exposed. (**c**) Cores were taken towards the left of the image in karst holes, the site was submerged at all times. (**d**) Cores were taken towards the bottom of the image while the area was completely exposed at low tide. (**e**) cores were taken towards the middle of the image, submerged in shallow water.

Sediment push cores were taken at four sampling sites across the lagoon and the main island Grand Terre (Fig. 1). Samples were taken during the day at low tide.

**Figure 2:**
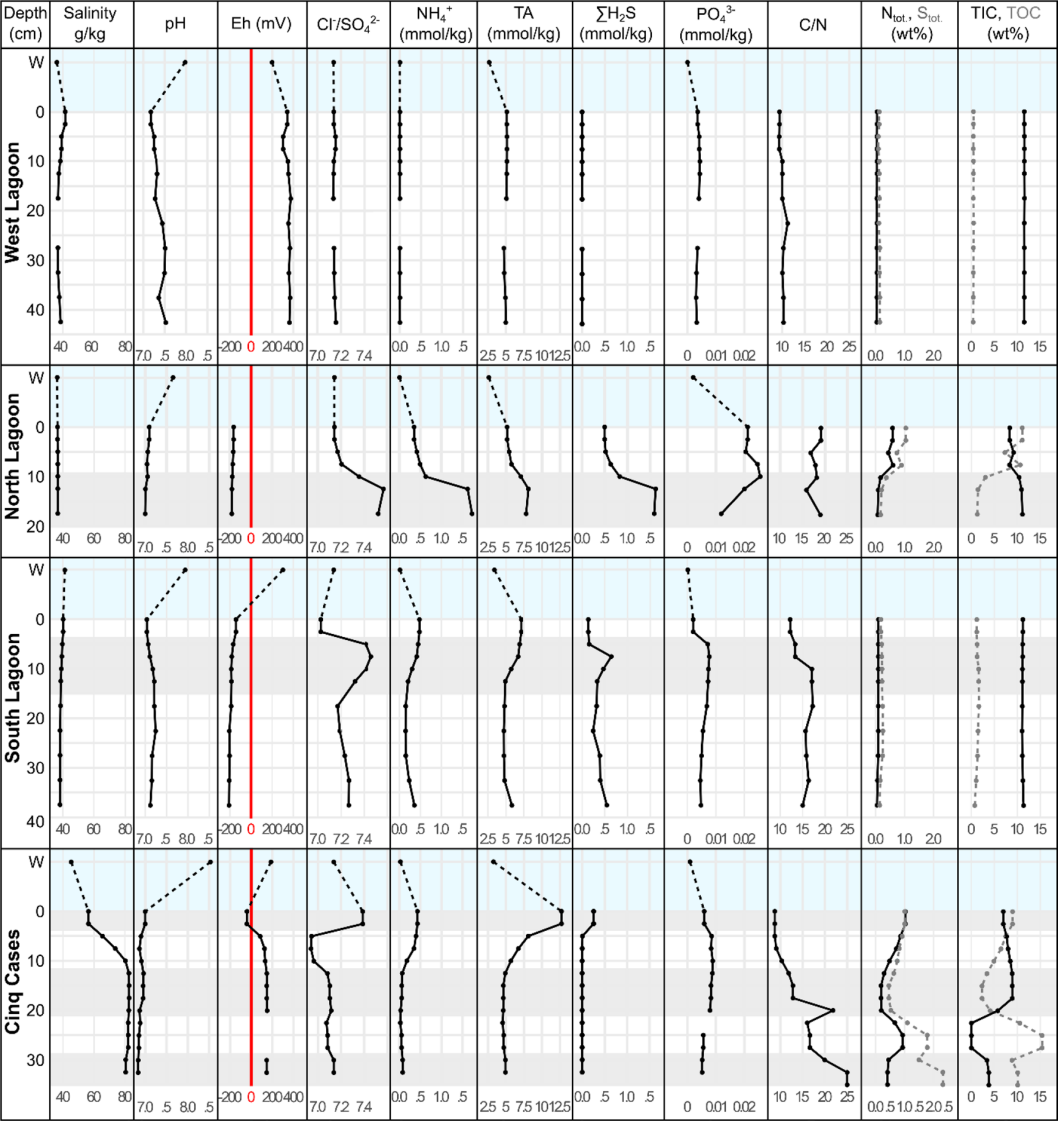
Selected porewater and bulk sediment geochemical profiles of the West Lagoon (WL), North (NL) and South (SL) Lagoon, and Cinq Cases (CC). Porewater profiles and sediment geochemistry were measured in two separate cores, except at Cinq Cases, where the same core was used. The water column (W) is set apart from the sediments by dashed lines and blue colour. Grey and white backgrounds indicate the geochemical zonation of the cores. The red line indicates the transition from reducing to oxidizing redox (Eh) conditions. Measurement accuracy deviated by less than 3%.

All cores were limited in depth by the underlying karstic limestone. None showed the expected characteristic lamination observed by Gaillard et al.^21^. Porewater geochemistry and bulk sediment geochemistry were measured in 2.5–5 cm intervals at each sampling site (Fig. 2, Supplementary Table S1) and correlated against depth using Spearman-Rank correlations (Supplementary Table S2, Supplementary Fig. S1).

In the lagoon-derived cores, pore water salinity ranged from 36 to 41 g/kg at low tide, while Cinq Cases ranged between 42 and 82 g/kg. Changing sulphate concentrations in relation to salinity are shown as SO_4_^2−^/Cl^−^ ratios.

The West Lagoon site was characterized by fine carbonate sand (Fig. 1b, Table 1) covered by a pink and green, slightly lithified top layer of 2–3 cm. The cores were sampled at low tide when the sand was completely exposed.

**Table 1:** Sediment characteristics (a) and sequencing features (b) of all sampling sites.

Location	West Lagoon		North Lagoon		South Lagoon		Cinq cases	
**(a) Sediment cores and features**								
Mean depth (cm)	40.8		15.8		34.2		27.5	
Latitude	− 9.44733		− 9.38396		− 9.44298		− 9.42979	
Longitude	46.23641		46.36124		46.39179		46.49524	
*Sediments*	Carbonate sand		Coarse carbonate sand and silt		Fine-grained carbonate mud		Fine-grained carbonate silt to mud	
Redox conditions	Oxidizing		Reducing		Reducing		Reducing to oxidizing	
Porewater exchange	Tidal porewater exchange		Advection, bioturbation		Advection, bioturbation		Evaporation, diffusion	
Additional features	Lithified surface layer with biofilm		Rich in organic matter and faeces from bird colony		0–15 cm highly bioturbated; 15-bottom high plant detrital input		Surface covered by microbial mat, strong salinity and C/N gradient	

Location	West Lagoon		North Lagoon		South Lagoon		Cinq Cases	
Material	DNA	RNA	DNA	RNA	DNA	RNA	DNA	RNA
**(b) Sequencing features (Mean)**								
Samples (N)	35	46	23	25	35	36	69	73
Raw reads	62,468	66,243	54,961	104,909	59,381	90,070	68,990	61,756
Processed reads	50,923	55,880	43,171	71,206	48,033	63,605	56,897	45,000
Total ASVs	11,731	8,555	13,989	12,831	13,335	11,441	16,393	14,561
Unclassified taxa (%)	9.5	2	13.4	10	4.1	3.5	17.1	8.7
Shannon (H’)	3.99	2.17	4.86	4.23	3.97	3.05	4.82	4.12
Faith’s PD	230.9	94.6	350.1	236.5	254.7	144.5	322.8	207.1
Chao1	2147.4	659.8	4026.5	2770.7	2420.3	1451.1	2230.3	1451.2

(a) The main environmental characteristics for each sampling site, including sediment type and depth, redox conditions, and mode of porewater exchange.

(b) total sample number and ASV counts, mean percentage of unclassified taxa and average diversity indices between the sampling sites and community fractions.

Data were rarefied at 19,906 reads per sample for calculation of diversity indices. The phylogenetic diversity is based on a midpoint-rooted phylogenetic tree.

The tides drain and flush the porewater space with fresh seawater. Oxidizing conditions with a mean Eh of + 340 mV and uniform distributions of total alkalinity (TA) and SO_4_^2−^/Cl^−^ prevailed throughout the West Lagoon sediment column (Supplementary Table S1). Ammonia concentrations (NH_4_^+^) did not exceed 1.5 µmol kg^−1^ and total sulphide (ΣH_2_S) was below detection limit of 0.5 µmol kg^−1^ (Fig. 2). All other geochemical measurements remained stable throughout the West Lagoon sediment and water column (Fig. 2). The North Lagoon cores were sampled near a red-footed booby (*Sula sula*) colony (Fig. 1c, Table 1), leading to increased PO_4_^3−^ concentrations due to their droppings (Fig. 2, Supplementary Table S1). The sediment was covered by water and consisted of fine to coarse carbonate mud, silt, and shell debris with fully reducing conditions. Below a surface zone (0–7.5 cm), a significant increased total alkalinity (TA), NH_4_^+^, and ΣH_2_S and a decrease in SO_4_^2−^/Cl^−^ marked a sulphate reduction zone (10–17.5 cm bsf; Fig. 2, Supplementary Fig. S1, Supplementary Table S1). The South Lagoon sediment was sampled near the highly bioturbated mangrove edge on a large exposed tidal flat. The sediment consisted of up to 40 cm deep fine-grained, grey carbonate mud (Fig. 1d, Table 1) with an Eh around -200 mV. Following a surface zone with high SO_4_^2−^/Cl^−^ ratio from 0 to 2.5 cm below the surface (cm bsf), an enhanced sulphate reduction zone with low SO_4_^2−^/Cl^−^ and a peak in ΣH_2_S was observed between 5 and 15 cm (Fig. 2). The bottom 15–40 cm bsf were traversed by mangrove roots and debris, which explain the increase in C/N from marine values around 10 to more terrestrial values above 15^29^ (Fig. 2, Supplementary Table S1). SO_4_^2−^/Cl^−^ increased and ΣH_2_S decreased in this zone, and TA and NH_4_^+^ significantly dropped in concentration, suggesting diminished levels of sulphate reduction and ammonification. The Cinq Cases pool system on the island of Grand Terre (Fig. 1e, Table 1) is dependent on meteoric water during the rainy season (November–April) and occasional flooding from the lagoon during spring tides. Some areas experience minor tidal water level fluctuations, suggesting low connectivity to marine waters through the karstic limestone^26^. The sediment was covered by a 1–2 cm thick microbial mat with peaks of SO_4_^2−^/Cl^−^ ratio, ΣH_2_S, TA and NH_4_^+^ at the mat bottom. The underlying sediment consisted of light grey silt and showed a considerable decrease in concentration of the latter porewater components and an increasing SO_4_^2−^/Cl^−^ ratio. The drop occurred alongside a significant increase in salinity (*P* < 0.05) from 56 g/kg in the mat to 82 g/kg at 10 cm bsf (Fig. 2, Supplementary Tables S1 and S2). The Eh increased from reducing conditions (− 41 mV at 2 cm bsf) to increasingly oxidizing conditions, stabilizing at + 150 mV around 10 cm bsf. Below 10 cm of depth SO_4_^2−^/Cl^−^ recovered to values around 0.141 and the other porewater components remained stable while ΣH_2_S was below detection limit. A further change in sediment stratification was observed as a high TOC layer between 22.5 and 27.5 cm bsf. TIC was almost completely absent in this layer, which had an ochre colour. Total N and S in the sediment matched the peak in TOC (Fig. 2). The C/N ratio increased significantly with depth (Fig. 2, Supplementary Fig. S1), culminating in a dark brown sediment layer (Fig. 1e) rich in plant-based organic matter.

### Bacterial community composition and diversity in sediments of Aldabra

**Figure 3:**
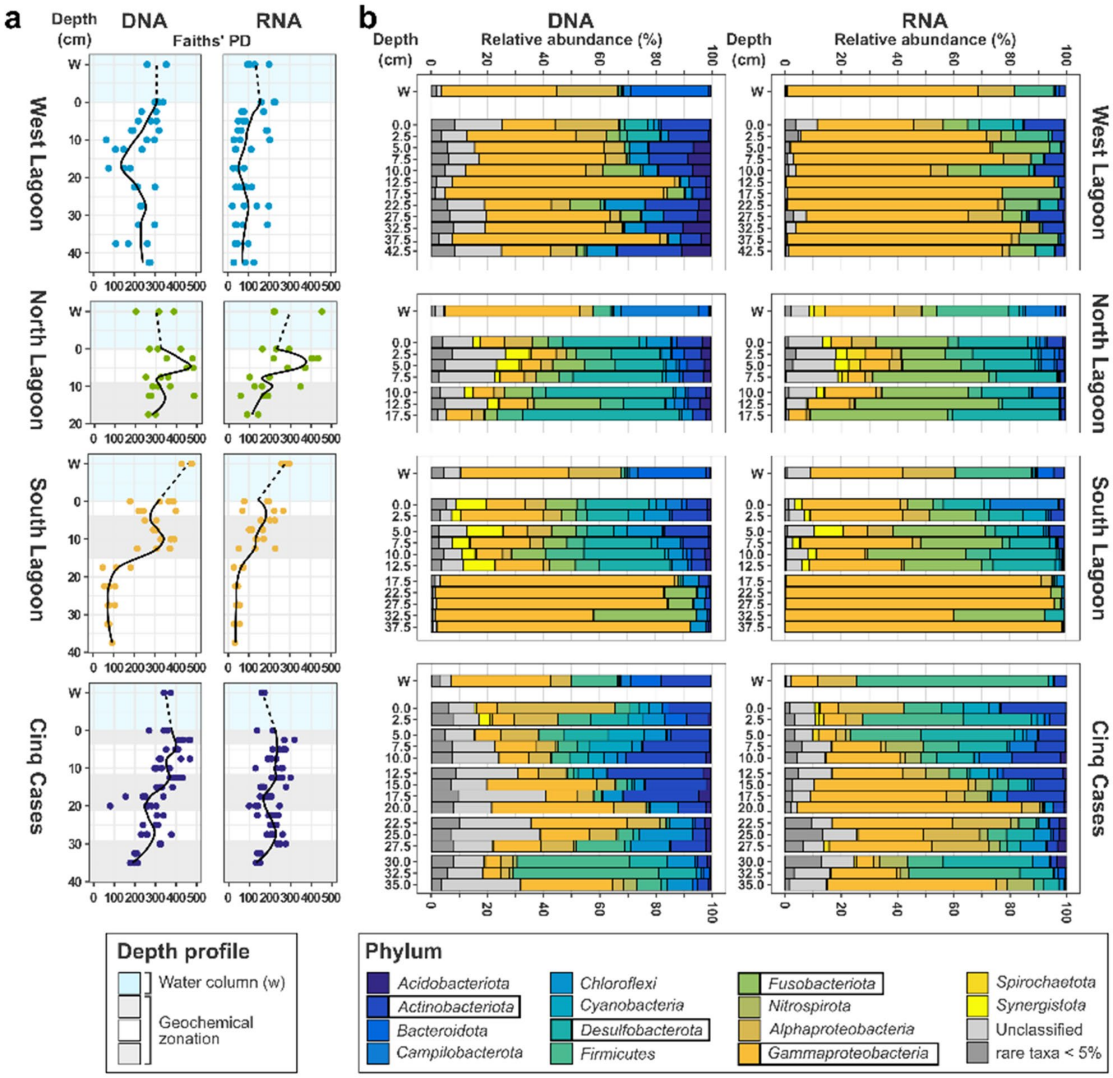
Overview of phylogenetic diversity gradients (**a**) and bacterial community composition (**b**) along sediment cores of the Aldabra Atoll. (**a**) Faiths’ phylogenetic diversity (PD) of the DNA and RNA-based community in relation to depth at each sampling site. (**b**) Most abundant bacterial phyla (> 5% relative abundance in at least one sample) in the total (DNA) and potentially active (RNA) community of the sediment and water column (W). Phyla below the threshold are summarized as rare taxa. Replicate samples from each core were averaged by depth. The most abundant phyla are highlighted by black boxes in the legend. Shading (**a**) and gaps (**b**) highlight the geochemical zonation.

Sequencing of the V3-V4 region of 16S rRNA genes (total or DNA-based community) and transcripts (active or RNA-based community) yielded a total of 8,473,178 (DNA) and 9,941,279 (RNA) high-quality reads. On average 79% of the reads passed bioinformatic processing and taxonomic assignment (Table 1, Supplementary Table S3). The final amplicon sequence variant (ASV) count was 32,331 in the total and 28,212 in the active community. Bacterial diversity and richness were significantly higher in the total than in the active community (Mann–Whitney U: W_PD_ = 21,506, p < 0.001; W_chao1_ = 20,443, p < 0.001), which is also reflected in the lower proportion of unclassified taxa (Table 1). The average amount of unclassified taxa was highest at Cinq Cases for DNA-based (17.1%) and in the North Lagoon for RNA-based communities (10%) (Table 1). Faith’s phylogenetic diversity (PD) ranged from 230.9 in the total and 94.6 in the active community of the West Lagoon to 350.1 (DNA) and 236.5 (RNA) in the North Lagoon (Table 1, Fig. 3). The richness indicator Chao1 followed the same pattern (Table 1). Shannon (H’), PD, Chao1 and species richness (SR) across all sampling sites decreased significantly with depth in both the DNA and RNA-based community (Supplementary Fig. S1). With exception of the PD, they correlate positively with TOC, N_tot_ and S_tot_ in the active but not the total community. The RNA/DNA-ratio is inversely correlated to diversity and richness.

In the West Lagoon, only bacterial richness in the active community decreased significantly with depth (Supplementary Fig. S1, Supplementary Table S2). The high diversity in the North Lagoon (0–7.5 cm bsf) occurred alongside increased phosphate and TOC concentrations and decreased in the sulphate reduction zone (10–17.5 cm bsf). In the South Lagoon, diversity and richness decreased significantly from the enhanced sulphate reduction zone to the underlying sediment (Fig. 2, Supplementary Fig. S1). At Cinq Cases the sulphate reduction zone and highest diversity coincided with the microbial mat (0–2.5 cm bsf). Below this zone diversity fluctuated alongside changes in SO_4_^2−^/Cl^−^ ratio, salinity, and TOC (Figs. 2, 3). Both DNA-based phylogenetic diversity and richness decreased significantly with sediment depth alongside extracted DNA concentrations (Supplementary Table S2).

### Total and active bacterial community structure along sediment cores

**Figure 4:**
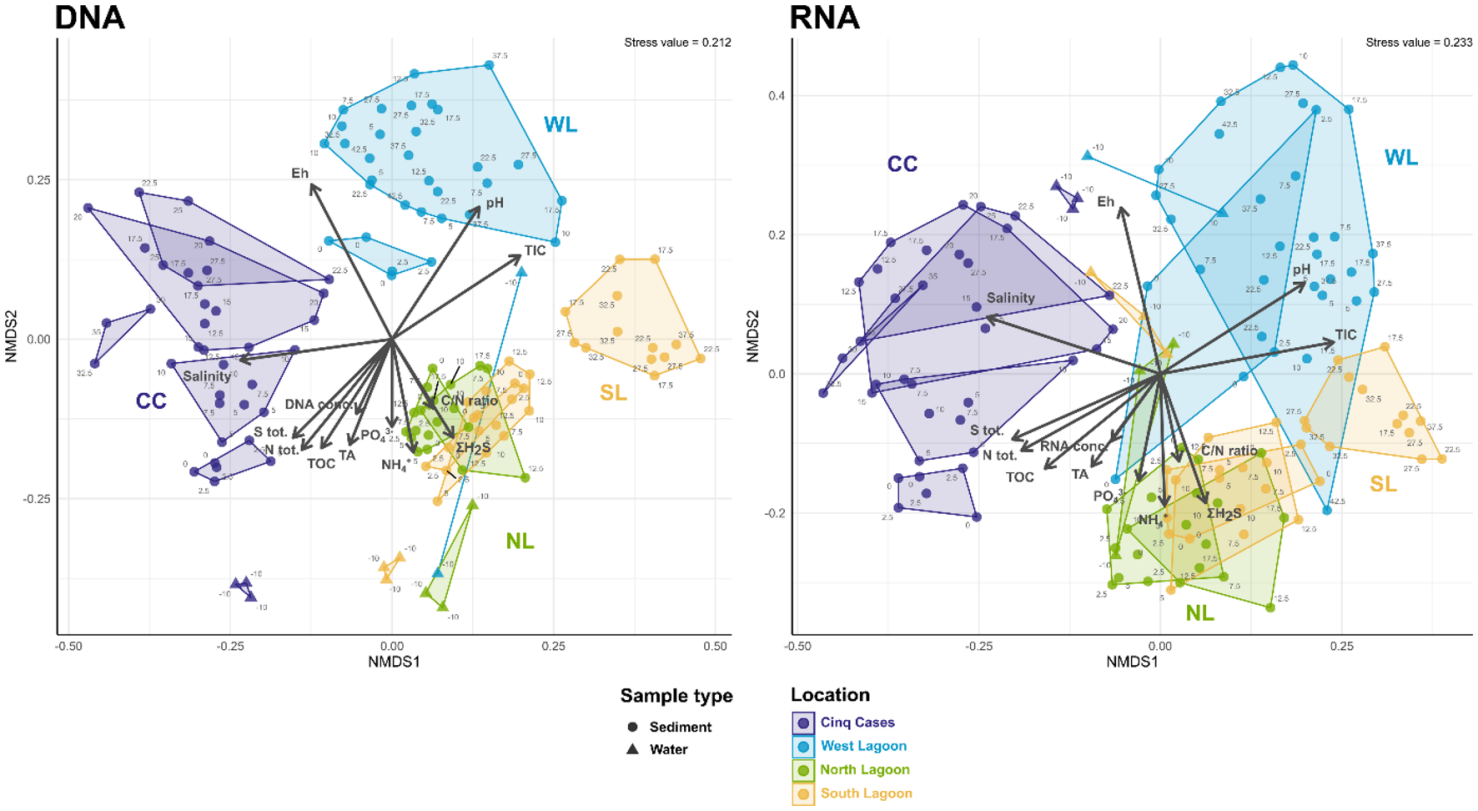
Non-metric multidimensional scaling of total and active bacterial community composition, geochemical zonation and significantly correlated environmental factors. NMDS based on a Bray–Curtis distance matrix of all DNA- and RNA-based sediment and water samples. The ASV table was normalized using GMPR^80^, and technical and biological replicates were averaged. Outlines indicate the geochemical zonation. Environmental fit was plotted if *P* ≤ 0.05 (Supplementary Table S2) using R^2^ as arrow length.

The compositions of the total and active bacterial community did not differ significantly from each other (Procrustes: correlation = 0.816, *P*-value = 0.001, n = 130; Fig. 4). The Cinq Cases community clustered in accordance with the geochemical zones determined by the porewater profiles (Fig. 2), while the surface and sulphate reduction zones of the North and South Lagoon overlapped. The sediment crust in the West Lagoon (0–2.5 cm bsf) was treated as individual zone and clustered separately in the total, but not in the active community (Fig. 4). Environmental fit shows key factors which significantly correlate with the community ordination. The highest extracted DNA and RNA concentrations, TOC, N_tot_, S_tot_ and TA are linked to shallow sediment depth. TIC and pH correlate with the West Lagoon sediment. The North and South Lagoon surface and sulphate reduction zones correlate most with ammonia, ƩH_2_S and C/N ratio. Salinity is correlated to the Cinq Cases community. The Eh correlates with the deeper sediment of the West Lagoon and Cinq Cases.

**Figure 5:**
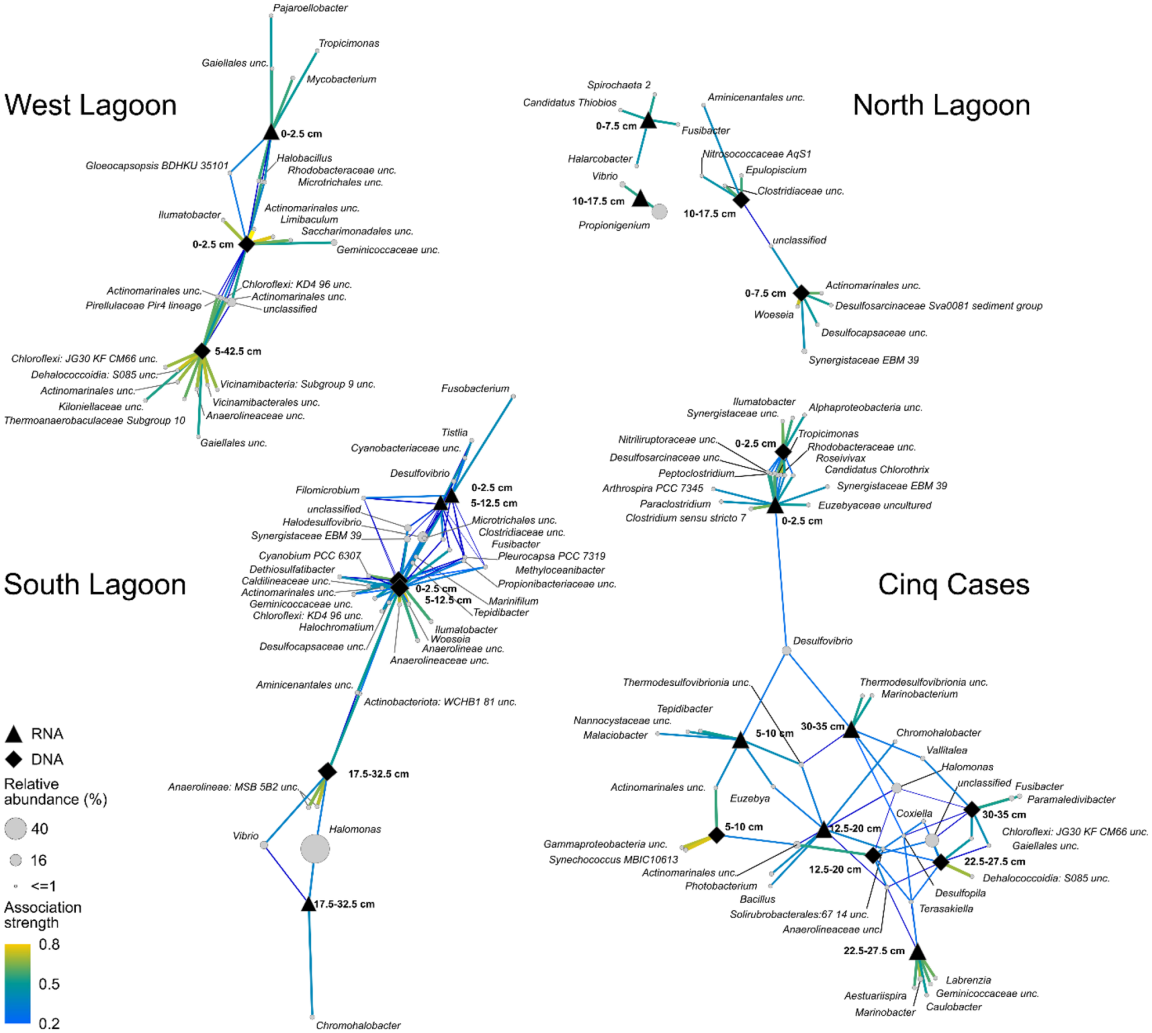
Genera associated with the total or active community fraction at each sampling site based on the geochemical zonation. The network was calculated using the multipatt analysis from the indicspecies package^88^. Circle sizes indicate the mean relative abundance between all samples. Edges are coloured according to their association strength to each target zone. Unclassified signifies ASVs which could not be taxonomically classified at genus level.

The most abundant bacterial phyla (Fig. 3) and genera (Supplementary Fig. S2) highlight broad shifts in community composition along the sediment cores. Association networks were calculated to identify key genera which preferentially occur within the total or active community and each geochemical zone (Fig. 5, Supplementary Fig. S3).

The West Lagoon sediment was dominated by *Gammaproteobacteria* (*Pseudomonas*), which were also detected in some samples within the water column (Fig. 3, Supplementary Fig. S2). As they were present at all depths *Pseudomonas* members were not significantly associated with a particular sediment zone (Fig. 5). The top 0–2.5 cm bsf consisted of a lithified crust, with high relative abundances of *Alphaproteobacteria* and *Halodesulfovibrio* (Figs. 3, 5), and was considered as a separate layer in the subsequent analysis. The crust was associated with *Rhodobacteraceae* and cyanobacterial *Gloeocapsopsis*, as well as uncultured *Actinomarinales* at the total and active community level. *Tropicimonas* and three other genera were associated only with the active community. The total community of the crust had the highest amount of rare associated genera at the West Lagoon (Supplementary Fig. S3). The association index did not identify any abundant or rare genera associated with the active community at 5–42.5 cm bsf. The total community in these sediments was associated with *Chloroflexi* and *Thermoanaerobaculaceae* (Fig. 5).

The North Lagoon cores exhibited the highest bacterial diversity and richness (Table 1, Fig. 3a), its community shifting gradually with depth. *Desulfobacterota*, particularly *Halodesulfovibrio*, occurred up to 52% relative abundance at DNA level and *Fusobacteriota* up to 49% at RNA level (Fig. 3, Supplementary Fig. S2), both increasing with sediment depth. At RNA level, the genus *Propionigenium* was strongly associated with the sulphate reduction zone at 10–17.5 cm bsf (Fig. 5) in which it was the most abundant genus. *Campylobacterota* (Fig. 3) were present within the water column (uncultured *Arcobacteraceae*) and throughout the sediments (*Halarcobacter*, *Sulfurimonas*) (Fig. 5, Supplementary Fig. S2). *Halarcobacter* was also associated with RNA-based community at 0–7.5 cm bsf (Fig. 5).

There was no clear community shift from surface to sulphate reduction zone in the South Lagoon. The phylum and genus level community composition mirrored that of the North lagoon (Fig. 3, Supplementary Fig. S2) and clustered closely together (Fig. 4). The fusobacterial *Propionigenium* was present throughout the sediment, particularly in the active community (Supplementary Fig. S2). Most associated taxa were shared between surface and sulphate reduction zone. *Cyanobacteria* were more strongly associated with the total than the active community. A strong shift in bacterial community composition occurred at 17.5 cm bsf accompanied by a drop in phylogenetic diversity (Fig. 3). From 17.5 cm bsf, *Halomonas* contributed up to 87% of the total and 66% of the active community (Supplementary Fig. S2). The number of associated bacterial genera decreased to three in the RNA-based and four in the DNA-based community, namely *Chromohalobacter* (RNA), two uncultured *Anaerolineae* (DNA), *Halomonas* and *Vibrio* (shared) (Fig. 5). The same trend was observed for the fraction with relative abundances < 2% (Supplementary Fig. S3).

The sediment at Cinq Cases showed the strongest changes in community along the sediment column. Starting in the microbial mat and underlying sediment (0–2.5 cm), the community shifted from abundant *Alphaproteobacteria* (*Tropicimonas*) to *Cyanobacteria* (*Synechococcus*), *Desulfobacterota* (*Desulfovibrio*) and increasing proportions of *Actinobacteriota* (*Actinomarinales*) at 5–10 cm bsf (Figs. 3, 5). *Cyanobacteria* peaked in the DNA-based community at 5–10 cm bsf, while the RNA-based community harboured increased proportions of *Campylobacterota* (Fig. 3b). *Actinobacteriota* and *Gammaproteobacteria* (*Halomonas*) were most abundant in the 12–20 cm bsf zone (Fig. 3) where some of the lowest values for diversity were recorded (Fig. 3a). Higher relative abundances of *Chloroflexi* (DNA) and *Alphaproteobacteria* (RNA) were observed from 22.5–27.5 cm bsf alongside a peak in TOC and increasing phylogenetic diversity (Fig. 3a). The bottom sediment zone (30–35 cm bsf) was characterized by a high proportion of *Firmicutes* and *Desulfobacterota* (Fig. 3b). The association network for Cinq Cases showed that the total and active community at 0–2.5 cm bsf shared most of their associated genera (> 5%) (Fig. 5). A larger number of rare genera (< 5%) was connected with the total (152) than with the active (54) community (Supplementary Fig. S3). At 5–10 cm bsf the campylobacterial *Malaciobacter* was associated with the total, while *Synechococcus* MBIC10613 was strongly associated with the active community. Alongside the low diversity, low TOC, total S and N (Fig. 2), a low number of genera were detected in association with the rare community from 12 to 20 cm bsf (Supplementary Fig. S3). The active community at 22.5–27.5 cm bsf was the most distinct from other sediment layers at Cinq Cases. It was associated with *Alphaproteobacteria* (e.g., *Aestuariispira, Labrenzia, Caulobacter*) and *Marinobacter* (*Gammaproteobacteria*; Fig. 5). The total community at this depth showed a preference of S085 *Dehalococcoidia*. The sediments from 30 to 35 cm bsf were associated with *Fusibacter* in the total, and *Desulfovibrio* and uncultured *Thermodesulfovibrionia* in the active community (Fig. 5, Supplementary Fig. S2)*.* The association network of rare genera (Supplementary Fig. S3) generally shows much less interconnectivity between the deeper sediments, than the network of most abundant genera (Fig. 5).

Despite their spatial proximity, the bacterial community observed in the water samples differs strongly from the sediment communities. The amount of unknown and rare bacterial taxa within the water samples was low with < 9% relative abundance in the total and < 7% of the active community (Fig. 3b). Of the overall 30 most abundant genera, only few appeared in the water column with low relative abundances (Supplementary Fig. S2). In addition to the ubiquitous *Gammaproteobacteria* (*Litoricola*), the water column harboured high abundances of *Alphaproteobacteria* (HIMB11) and *Bacteroidota* (uncultured *Cryomorphaceae*) at total community level (Fig. 3, Supplementary Fig. S2). *Bacteroidetes* reached only low relative abundances in the active community, while *Firmicutes* were more abundant.

## Discussion

While we did not observe the expected pure laminated mud on Aldabra^21^, we recovered distinct sediment cores from four different locations on the atoll. This provided us with an ideal opportunity to study bacterial succession and geochemical zonation under changing porewater dynamics and different sediment settings. Due to the high tidal amplitude^20^ and resulting strong tidal currents, we found sediments to poorly accumulate in the lagoon, limited in depth by the karst structure of the limestone below. Nevertheless, we expected to find bacterial communities and porewater gradients reflecting the standard geochemical zonation. This would follow an energetically favourable progression from oxygen to nitrate to sulphate as main electron acceptor^2^. Our results, however, show overlapping signatures of porewater dynamics, location and time which diverge from this pattern.

**Figure 6:**
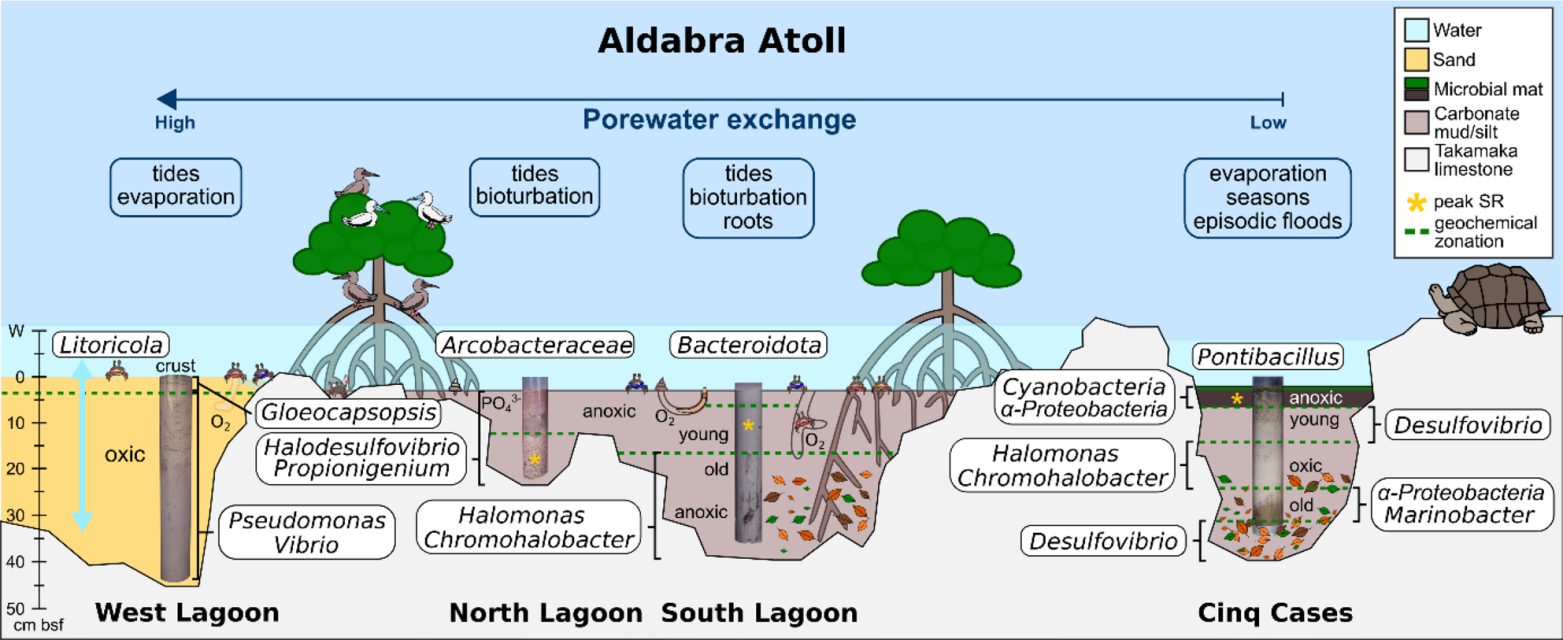
Model of Aldabra’s sediment environments, detected bacterial key taxa and geochemical zones. The sampling sites are ordered by sediment dynamic from left to right, starting with the highest. Most abundant bacterial taxa at each site are indicated close to the respective sediment core. Pictograms indicate further factors, such as macrofaunal input, bioturbation and mangrove roots and detritus.

A gradient of decreasing porewater dynamics can be observed from the West Lagoon, over the North and South Lagoon, to Cinq Cases (Fig. 6). It is linked to the magnitude of porewater exchange due to different porosities of the fine to coarse grained sediments and tidal impact. In the West Lagoon the porous, and hence highly permeable, carbonate sand falls dry at low tide leading to the exchange of porewaters visible from the absence of clear shifts in the porewater chemistry and bacterial community profiles. Tidal porewater exchange also leads to an overlap of water and sediment communities of the West Lagoon (Fig. 4, Supplementary Fig. S2) and similarly low diversity (Fig. 3a). This suggests that the bacterial community is at least partially tidally exchanged. Planktonic taxa which are associated with the water column, i.e., *Litoricola* and alphaproteobacterial HIMB11^30,31^, may be removed and replenished with the porewater and do not accumulate in the sediment, which has also been observed elsewhere^32^. As the cores were sampled during low tide, the sediment data most likely depicts a combination of particle or sand-grain associated bacteria, which can comprise up to 42% of the bacterial community^33^ and taxa percolating through the sediment from the hinterland. At the sediment surface of the West Lagoon a lithified crust was observed (0–2.5 cm bsf) covered by a thin pink and green biofilm. Its community composition and crust lamination resemble microbial mats in other tropical locations^16,34^. Only the uppermost sample of the active community reflects the current biofilm where photosynthetic *Gloeocapsopsis* are potentially involved in the lithification process of the crust^35^. Amongst other bacteria, the total crust community is inhabited by *Desulfobacterota*, *Chloroflexi* (Figs. 3b, 5) and diverse rare taxa (Supplementary Fig. S3). These may represent remnant members of a microbial mat or biofilm, as only few of these occur in the active community. When the mat dried out and calcified, their DNA may have been protected by exopolymeric substances in the surrounding mat^6^.

Like the ooid sediments of the Bahamas^36^, the Kiritimati lagoon^15^, Californian and Hawaiian sands^32,37^, the remaining sand community of the West Lagoon is dominated by *Gammaproteobacteria*, primarily *Pseudomonas* and *Vibrio* (Fig. 3b, Supplementary Fig. S2). *Pseudomonas* have previously been observed associated with Hawaiian sand above the high tide mark^37^ and overrepresented in sand column flowthrough in California^32^. This observation was linked to a closer connection to soil as common source for *Pseudomonas*^37^. The higher than marine salinity (> 3.5 g/kg) in the cores surface and bottommost sediments shows that evaporated mangrove water is dragged in through the sediment. *Pseudomonas* may therefore originate from the mangrove hinterland. However, no data are currently available to confirm this. Further, their high relative abundance may be exaggerated due to the overall low biomass in the sand and differences in extraction efficiency between the sand and other sediments. The total community of the sand (5–42.5 cm bsf) also shows traces of anaerobic digesters, such as *Anaerolinea* or *Thermoanaerobaculales* (Fig. 5). They may have accumulated through tidal porewater drag from the mangrove hinterland and are either inactive under the oxidizing conditions or persist in oxygen-depleted micro-niches^36,38^.

Similar to the West Lagoon, *Bacteroidota* are most abundant in the total community of the North and South Lagoon water (Fig. 3b). As globally abundant primary degraders of phytoplankton blooms^39,40^, their activity may be linked to phytoplankton abundance on Aldabra. Blooms may occur in eddies around the atoll^41^ or more stagnant areas in the lagoon. Site specific differences in the lagoon water were most obvious in the North Lagoon where the presence of a bird colony led to increased phosphate concentrations (Fig. 2) and an enrichment of *Campylobacterota* (Fig. 3b) including uncultured *Arcobacteraceae* and *Arcobacter*, which are typical for faecal contamination^42^*.* The North and South Lagoon sediment represent environments with intermediate porewater exchange (Fig. 6). The sediments do not fall completely dry at low tide and are affected by superficial mixing through the currents and bioturbation. The North Lagoon sediments consist of course sand, silt, and shell debris, with limited porewater exchange due to their position in karst holes and narrow depressions of the lithified Pleistocene limestones. Whereas the South Lagoon resembles the pure mud observed by Gaillard et al*.*^21^ most closely, however lacking lamination. Nevertheless, the porewater profiles of the two sites both initially follow the standard geochemical zonation^2^ with a surface zone of up to 7.5 and 2.5 cm in the North and South Lagoon respectively, and an enhanced sulphate reduction zone below. In the surface zone, we detected typical photo(hetero-)trophic *Cyanobacteria* (*Pleurocapsa, Cyanobium*) and *Alphaproteobacteria* (*Tropicimonas, Roseivivax*) indicative of remnant oxygen and light exposure. As oxygen is usually consumed within the first millimetres of sediment^43^, they occurred alongside bacteria known for their anaerobic metabolism in bioturbated sediments (*Propionigenium, Synergistaceae*)^44^ and sulphate reduction ((*Halo-*)*Desulfovibrio*) (Figs. 3, 5, Supplementary Fig. S2). Benefiting from the sulphate reduction in the zone below, *Halarcobacter*^45^ and *Candidatus* Thiobios^46^ likely oxidize sulphur compounds in the sediment directly above the highest measured ΣH_2_S concentrations (Fig. 5). The transition to the sulphate reduction zone can be identified through a significant decrease in SO_4_^2−^/Cl^−^ ratio, and increase in ΣH_2_S and TA, respectively (Fig. 2, Supplementary Fig. S1). Decreasing SO_4_^2−^/Cl^−^ ratio and higher ΣH_2_S are linked to increasing relative abundances of (*Halo-)Desulfovibrio* and *Desulfocurvus* (Supplementary Fig. S2), which belong to the most prominent phylum of sulphate reducers, *Desulfobacterota*. The relative abundance of proteolytic *Synergistia* (EBM 39) increased alongside *Desulfobacterota*, particularly in the active community. These *Synergistia* may provide additional sulphate and short-chained fatty acids for the ongoing sulphate reduction^47^. The increase in TA and NH_4_^+^ are likely linked to higher fermenting activity by abundant *Propionigenium*^48^ in addition to other anaerobic fermenters. While the porewater profiles show a distinct separation of biogeochemical zones, the bacterial community transitions more gradually resulting in a mixed surface and sulphate reduction zone (Fig. 3b). The zone may be caused by strong bioturbation and a certain degree of tidal mixing of the fine-grained sediment. This results in a multitude of micro-niches, which can sustain a metabolically diverse bacterial community^5^. Regardless of the drop in SO_4_^2−^/Cl^−^ ratio and peaks in TA, the bacterial diversity in these sediments peaks within the first 10 cm of depth. This supports the observation that diversity itself is determined more by depth than the geochemical zone^12^. Observed correlations of diversity with NH_4_^+^ in the North Lagoon, are therefore likely not causative. Bacterial ammonia oxidizers were rarely detected, yet ammonia-oxidizing archaea may be prevalent near the sediment surface^49^. The high phylogenetic diversity and variety of metabolic groups in these sediments, can be linked to both the porewater exchange with the water column^4^ and strong bioturbation by i.e., fiddler crabs^5^, which introduce nutrients and oxygen. The mixing creates a multitude of microinches in the mainly anoxic sediments, leading to an enrichment of rare bacterial genera (Supplementary Fig. S3). This results in the observed mixed surface and sulphate reduction zone of the bacterial community, which cannot be distinguished as clearly as the porewaters imply.

While the North Lagoon sediments end with the sulphate reduction zone, the South Lagoon shows an additional atypical zone below. The change occurs below 17.5 cm where low quantities of extractable DNA and RNA, and significantly lower bacterial diversity and richness indicate low microbial biomass (Fig. 3, Supplementary Table S2, Fig. S1). A decrease in TA and NH_4_^+^ also suggest lower heterotroph activity in the sediment (Fig. 2). Typical sulphate reducing taxa, such as *Halodesulfovibrio*, are virtually absent from this sediment zone, explaining the increase in SO_4_^2−^/Cl^−^ ratio. Although the presence of methanogenic archaea cannot be excluded, the abundant aerobic and facultatively anaerobic *Halomonas*, *Vibrio* and *Chromohalobacter*^50–52^ do not match our expectations for anoxic sediments. The porewater profiles do not provide indications for the cause of the low diversity. The bulk sediment data shows an increase in C/N (Fig. 2), which can be linked to the onset of mangrove detritus also visible in the core images (Supplementary Fig. S4). This is the only recorded change in the measured sediment data. We hypothesize that the change of the bacterial community is linked to the onset of mangrove detritus. The detritus may be in various states of decomposition and provide a more complex substrate for degradation. At this stage, the majority of remnant DNA from previous inhabitants may already be degraded, removing most rare and sedimented taxa (Fig. 5, Supplementary Fig. S3). Deeper sediment layers have previously been found to record generalist communities, which can persist on less labile substrates through their efficient energy metabolism^13,53^. Even though the age difference is short on a geological time scale, this would explain the high relative abundances of a few versatile and specialised genera. The available mangrove detritus may be in various states of decomposition visible through the increase in C/N ratio (Fig. 2)^54^. As both *Halomonas* and *Chromohalobacter* have been observed to break down even aromatic hydrocarbons^55^, they may thrive on the complex mangrove detritus. Other taxa, such as uncultured *Anaerolineae* may follow a different strategy and persist in this sediment through a reduced metabolism and specialisation for starvation^56^. This would also explain their reduced abundance at active community level. Deeply burrowing marine fauna or plant-degrading fungi may also impact the older sediment^54,57^.

At the first glance, Cinq Cases is the most settled of the four settings regarding the exchange of porewaters. It is barely affected by tidal fluctuations, therefore time and season become dominant factors. With exception of a few disturbances by giant tortoises, the sediment is not bioturbated. The water level at Cinq Cases is mainly determined by the rainy season and high spring tides, as well as strong evaporation during the dry season^26^. This has allowed the accretion of sediments over time, creating five distinct sediment layers including a microbial mat on the surface (Fig. 6). We expected to see clear porewater zones and corresponding community shifts in the cores, together with fully reducing redox conditions. On the contrary, we encountered mainly oxidizing sediments with strongly overlapping signatures. Starting at the sediment surface, the microbial mat drives the porewater geochemistry of the first few centimetres. It harbours the main sulphate reduction and ammonification zone of the sediment, indicated by a drop in SO_4_^2−^/Cl^−^ ratio, peaks in ΣH_2_S, ammonia and negative Eh (Fig. 2). The bacterial community composition resembles previous studies of microbial hypersaline mats and further sub-sectioning would likely yield a finer zonation^16,34^. The presence of *Synechococcus* in the total but not in the active community at 5–10 cm bsf (Fig. 5), denotes remnants of a former cyanobacterial bloom or mat. As a pile up of mats is often observed^16^ this sediment may represent the old sediment surface. Below the mat and down to a depth of 10 cm bsf, a strong salt diffusion gradient to moderately hypersaline conditions drives the porewater geochemistry (Figs. 2, 4). Simultaneously, the Eh changes to oxidizing conditions, a trend which cannot be explained by the measured porewater and bulk geochemical profiles. Based on the diffusion gradient and the change to oxidizing Eh, we suggest that a complete desiccation event has occurred in the past resulting in older evaporated sediment below 10 cm bsf. Like at the South Lagoon, we find a significant decrease in diversity and richness with depth (Fig. 3a, Supplementary Fig. S1), as well as *Halomonas* and *Chromohalobacter* associated with the active community below 12.5 cm bsf (Fig. 5). As mentioned above, these taxa may be adapted to a change to less labile organic matter and, in this case, salinity. As the C/N ratio significantly increases and the metabolic products of the fast-lived surface community become scarce, the ability to degrade complex organic matter may become an increasingly important driver for the Cinq Cases community. This culminates in a high TOC layer (22.5–27.5 cm bsf) where the typical carbonate particles (e.g., mollusc and green algal fragments, foraminifera) of Aldabra are absent. The layer harbours high abundances of aerobic *Alphaproteobacteria* (*Caulobacter*, *Aestuarispiira*) and *Marinobacter* (Fig. 5), which have been found in putrid^58^ and hydrocarbon-producing systems^59^, suggesting that they can degrade the organic matter responsible for the high TOC. *Caulobacter* have been reported to flourish in organic soils and survive desiccation^60^. As the Eh and salinity suggest the occurrence of a desiccation event (Fig. 2), this would explain their presence at this depth, as well as the overall low phylogenetic diversity (Fig. 3a). Slow growing *Desulfopila*^61^ and *Fusibacter* (*Firmicutes*), which have been found to occur in saline sediment with low sulphate reduction rates^62^, may survive through slower fermentation of a wide range of carbohydrates^63^. Alternatively, they may occur in micro-niches with their ideal living conditions, which cannot be assessed with the methods used in this study. This also applies to the bacterial community of the deepest sediment (30–35 cm bsf). Even though the porewaters suggest low sulphate reduction rates (Fig. 2), the bacterial community shows similarities to the uppermost 5–10 cm bsf, as the same sulphate reducing taxa are associated with the active community (Fig. 5). Notably, while the lagoon sediments harbour mainly *Halodesulfovibrio*, their environmental niche is filled by *Desulfovibrio* at Cinq Cases. The latter have been found in extreme hypersaline sediment and may be better adapted to saline conditions^64^. The higher abundance of *Fusibacter* in the total than in the active community, suggests that they only tolerate the suboptimal oxidizing conditions and persist in the sediment through a maintenance metabolism^53^ or in anoxic micro-niches. The change to more plant-bound organic matter (higher C/N and leaf litter) at this depth may again explain the change in community composition^54^, as well as the switch in dominant sulphate reducers throughout the cores (Fig. 5). Overall, the Cinq Cases profiles suggest, that desiccation events have caused overlapping signatures in both geochemical and bacterial community profiles, which are difficult to disentangle and beyond the scope of this study.

Regarding our initial research aims and hypotheses, we could not determine the bacterial communities and geochemical gradients analogue to former Jurassic lagoons, such as the Cerin, as comparable sediments could not be retrieved. Nevertheless, we were able to address key questions regarding the community composition, main geochemical gradients and likely causes for their transitions. Firstly, we could not confirm the assumption, that all sediment communities follow the typical biogeochemical zonation along electron transfer gradients. Peaks in SO_4_^2−^/Cl^−^ ratios, ƩH_2_S, TA and NH_4_^+^ were the main determining factors for geochemical zonation. Shifts in bacterial community composition occurred more gradually than in the porewater profiles, showing large overlaps between surface and sulphate reduction zone. Below the sulphate reduction zones, communities were characterized by low biomass, low diversity and less taxa known for their anaerobic metabolism. In agreement with^12,13^, this suggests that other factors than electron availability and the corresponding biochemical zonation have a larger impact on these sediment settings. We hypothesize that the changes in bacterial surface community composition are related to short-term porewater fluxes, while the availability of organic matter for degradation, alongside long-term seasonal and episodic changes drives the sediments below 10 cm.

Secondly, our sampling efforts confirm the hypothesis that bacterial diversity and richness significantly decrease with depth. Concomitantly we find that RNA/DNA ratios significantly increase with depth (Supplementary Fig. S1, Fig. 3a). This measure should be interpreted with care, due to a variety of confounding factors, including ribosomal content at different stages of cell development and dormancy^8^, as well as inaccuracies in spectrophotometric measurement. Nevertheless, the data support the observation, that the DNA-based community shows an overlap between current community members and eDNA, former, dormant, and sedimented taxa^6^. This applies particularly to the upper, more diverse sediment zones, which harbour a mixture of bacterial taxa in the total community, of which only few were found in the active community at the time of sampling (Fig. 5, Supplementary Fig. S2). With increasing depth, RNA/DNA ratios increase, suggesting that the total and active community overlap more closely, as fewer dead taxa and eDNA remain. While the analysis of total and potentially active communities provides a first understanding of the bacterial dynamics in relation to porewater data, they represent the initial step towards a closer understanding of the environment. Specific and selective analyses are required for accurate determination of bacterial metabolism in these sediments. These, however, may build upon the data we present here.

To conclude, this dataset represents the first in-depth assessment of multiple sediment cores from the Aldabra Atoll. As cross-sectional studies of this kind and sediment depth are scarce, the data demonstrate the diversity and variability that can be encountered within an atoll environment. The sediments characterised in this study did not show the anticipated typical lamination observed in the lagoon of the Cerin^21^. We therefore adjusted our approach to characterize each sediment profile individually. The bacterial communities and their diversity were placed into context with the geochemical profiles and their environment. We find that bacterial communities show alternate succession profiles as expected from standard biogeochemical zones^2^, whilst confirming the common trend of decreasing diversity with depth^12,13^. Comparison of both 16S rRNA genes and transcripts highlights where past and present bacterial communities may overlap. We hypothesize, that the tides, seasons, and availability of organic substrates may have a similarly large impact on the system, as the availability of electron donors. This study provides the basis for further in-depth analysis of specific sampling sites and individual members of the bacterial community.

## Methods

### Sample collection and storage

Samples were taken during an expedition to the Aldabra Atoll in November 2017. Sediments were sampled using push cores made from PVC tubes (Ø 63 mm, Thyssenkrupp Plastics, Essen, Germany) at three sites on the tidal flats of the lagoon (North, South and West Lagoon), and one site at the Cinq Cases pools on Grand Terre island (Fig. 1). At each sampling site, three sediment cores were taken for microbial analysis and one each for porewater and sediment analyses (Supplementary Fig. S4). The cores were subsampled at intervals of 2.5–5 cm bsf with the exclusion of 1 cm of the outer rim, to avoid cross contamination from the coring tube. Three 200 ml water samples were taken 10 cm above each sediment and filtered through a 0.2 µm polyethersulfone (Sartorius, Göttingen, Germany) and 3.0 µm polycarbonate (Merck, Darmstadt, Germany) filter sandwich with a diameter of 47 mm. Filter sandwiches were placed on NALGENE™ reusable filter holders with receivers and water samples filtered using vacuum from a NALGENE™ manually operated PVC Vacuum Pump (both Thermo Fisher Scientific, Waltham, MA, USA). All samples were immediately stored in RNAprotect™ Bacteria Reagent (Qiagen, Hilden, Germany). After transport, RNAprotect™ Bacteria Reagent was removed from all samples by centrifugation at 3,150 × *g* for 1 h. The supernatant was decanted, and samples were placed at -80 °C for long-term storage.

### Coextraction of DNA and RNA

Sediment samples were thawed on ice and homogenized before weighing into the extraction tubes. DNA and RNA were extracted simultaneously using the RNeasy™ PowerSoil Total RNA Extraction kit followed by the accessory RNeasy™ PowerSoil DNA Elution kit. 1 g of sediment or half of a filter (water samples) per extraction were used as recommended by the manufacturer (Qiagen, Hilden, Germany). Final elution was performed with 50 µl nuclease-free water (50 µl). In addition, RNA samples were supplemented with 1 µl of RiboLock RNase Inhibitor (Thermo Fisher Scientific, Waltham, MA, USA) before storage at -80 °C. DNA and RNA concentrations were measured using a NanoDrop 1000 (Thermo Fisher Scientific, Waltham, MA, USA).

### RNA purification and reverse transcription

Potential DNA contaminations were removed from RNA samples according to Schneider et al*.*^65^. RNA was purified using the RNeasy™ MinElute kit according to manufacturers’ instructions (Qiagen). Purified RNA was reverse transcribed using SuperScriptIV (Thermo Fisher Scientific) and the manufacturers’ instructions for gene-specific primers with the reverse primer S-D-Bact-0785-a-A-21^66^ as described in^67^. To inhibit RNases 1 µl of RiboLock (Thermo Fisher Scientific) was added to the reverse transcription reaction. cDNA was treated with 0.5 µl of RNase H (Thermo Fisher Scientific) for 20 min at 37 °C.

### Amplification and sequencing of bacterial 16S rRNA genes and transcripts

Bacterial 16S rRNA genes and transcripts were amplified by PCR using V3-V4 primers (SD-Bact-0341-b-S-17 and S-D-Bact-0785-a-A-21^66^, purified and sequenced as described by Berkelmann et al*.*^67^.

### Raw read and amplicon sequence processing

Raw reads were quality filtered and processed as described in detail by von Hoyningen-Huene et al*.*^68^. Where possible, processes were parallelized with GNU parallel 20,190,322^69^ and comprised the following steps and bioinformatic tools. Raw reads were quality-filtered with fastp 0.20.0^70^ and merged using PEAR v0.9.11^71^. Any remaining primer sequences were clipped using cutadapt 2.5^72^. After merging, sequences were processed into ASVs with VSEARCH v.2.14.1^73^. This included size-sorting, dereplication and denoising using the UNOISE3 algorithm^74^ and default parameters. Chimeras were removed using a de novo and reference-based search against the SILVA SSU 138 Ref NR 99 database^75^. Quality filtered reads were mapped back to the ASVs using *usearch_global* in VSEARCH. ASVs were taxonomically assigned using BLAST 2.9.0 +^76^ against the SILVA SSU 138 Ref NR 99 database with an initial minimum identity cut-off at ≥ 90%. Accession ID, % blast identity, % query coverage and e-value were retained for further qualit filtering steps. BLAST hits were deemed uncertain if the sum of BLAST identity and query coverage divided by two was ≤ 93%, as recommended by the SILVAngs guide^75,77^. Extrinsic domains (chloroplasts, mitochondria, Archaea, Eukaryota) were removed from the dataset. ASVs with a blastn identity below 95% were labelled “unclassified”. ASV tables were generated and formatted using biom tools v1.0^78^. ASVs were aligned using MAFFT v7.407^79^ and a phylogenetic tree was calculated using FastTreeMP 2.1.10^80^. The tree was midpoint-rooted using FigTree v1.4.4^81^. The fasta file including all ASVs can be found in Supplementary File S1 and the ASV table in Supplementary Table S4.

### Data analysis and visualisation

All data were analysed using R Version 4.0.0^82^ and RStudio Version 1.3.959^83^. ASV tables were normalized using two different methods depending on the analysis. GMPR was used as normalization for comparative analysis of the microbial community^84^ whereas diversity and richness indices were calculated from rarefied count data as recommended by Pereira et al*.*^85^. The former included the bar charts, heatmaps, NMDS and association networks. Data were analysed and visualized using *ampvis2*^86^, *vegan*^87^ and *ggplot2*^88^. The ASV count table was rarefied at 19,906 reads. The phylogenetic diversity (Faith’s PD) was calculated using *picante*^89^, a midpoint-rooted phylogenetic tree and the rarefied ASV table. Sediment zones were determined from the geochemical profiles (Fig. 2). Spearman Rank correlations were calculated on the sediment data and mean diversity and richness indices excluding the water column using the *Hmisc*^90^ package and visualised using *corrplot*^91^ with a significance cut-off of *P* < 0.01. Association networks for abundant and rare genera were calculated using the GMPR-normalized table of taxa above or below 2% (West, North, South Lagoon) or 5% (Cinq Cases) relative abundance. The association networks were calculated using the *indicspecies* package^92^ using multi-level pattern analysis (multipatt) with the “r.g” function. The geochemical sediment zones combined with total or active community were used as grouping variable for the analysis. The resulting network table was visualized using an edge-weighted spring-embedded layout in Cytoscape version 3.8.2^93^.

The Map of Aldabra, tidal tables and amplitudes were kindly supplied by the Seychelles Island Foundation (SIF). The global map indicating the location of Aldabra was generated using ArcGis Desktop 10.7.1.^94^ and Esri World imagery (Sources: Esri, DigitalGlobe, GeoEye, Earthstar Geographics, CNES/Airbus DS, USDA, USGS, AEX, Getmapping, Aerogrid, IGN, IGP, swisstopo, and the GIS User Community). Maps, photographs, and plots were combined using Inkscape 1.0^95^.

### Porewater and bulk sediment geochemistry

At each sampling site except Cinq Cases, one core was taken for bulk geochemistry and one for pore water chemistry in close lateral distance. The core at Cinq Cases was used for both porewater and bulk geochemical analysis. Each core was subsampled at intervals of 2.5–5 cm of depth. Redox potential (Eh) and pH were measured directly through boreholes in the cored sediments within 24 h after sampling with a portable WTW 340i pH meter, equipped with an Inlab Solids Pro pH-electrode (Mettler Toledo, Columbus, OH, USA) and a Pt 5900 A redox electrode (SI Analytics, Mainz, Germany; standard deviation ≤ 2%). Porewater was extracted from the cores with 5 cm CSS Rhizon samplers (Rhizosphere, Wageningen, Netherlands). Porewater alkalinity (TA), NH_4_^+^, PO_4_^3−^, ΣH_2_S, and total organic (TOC), total inorganic carbon (TIC), N, and S, were measured as described by Fussmann et al*.*^96^. Cations and anions were measured using ion chromatography (Metrohm 820 IC/Metrosep C3 and Metrohm 883 Basic IC/Metrohm A Supp 5, Metrohm, Herisau, Switzerland) with either suppressed or non-suppressed conductivity detection. Inductively coupled plasma mass spectrometry (ICP-MS) on an iCAP-Q spectrometer (Thermo Fisher Scientific) was used to measure Fe. Measured data had a standard deviation of ≤ 2% for IC and ≤ 3% for ICP-MS and were converted from molarity (mmol/l) to molality (mmol/kg) by density calculations with the PHREEQC software package version 3^97^. Photometric determination of nitrate failed in the field due to the very limited amount of extractable porewater. Further, concentrations were below the quantification limits of alternative ion chromatographic methods.

## Supplementary Information

Supplementary Information 1.

Supplementary Figure 1.

Supplementary Figure 2.

Supplementary Figure 3.

Supplementary Figure 4.

Supplementary Table 1.

Supplementary Table 2.

Supplementary Table 3.

Supplementary Table 4.

## Data Availability

All raw sequences were deposited at the NCBI Sequence Read Archive as part of the BioProject PRJNA611521 with the accessions SRR11295008- SRR1129550. An overview can be found in Supplementary Table S3.
