# Supplementary material for: DNA- and RNA-based bacterial communities and geochemical zonation under changing sediment porewater dynamics on the Aldabra Atoll
Source: Sci Rep. 2022 Mar 11;12:4257. doi: 10.1038/s41598-022-07980-0 (PMC8917147; doi:10.1038/s41598-022-07980-0)
Supplement: Supplementary file 2 — Supplementary Figure 1. [file 41598_2022_7980_MOESM2_ESM.pdf]

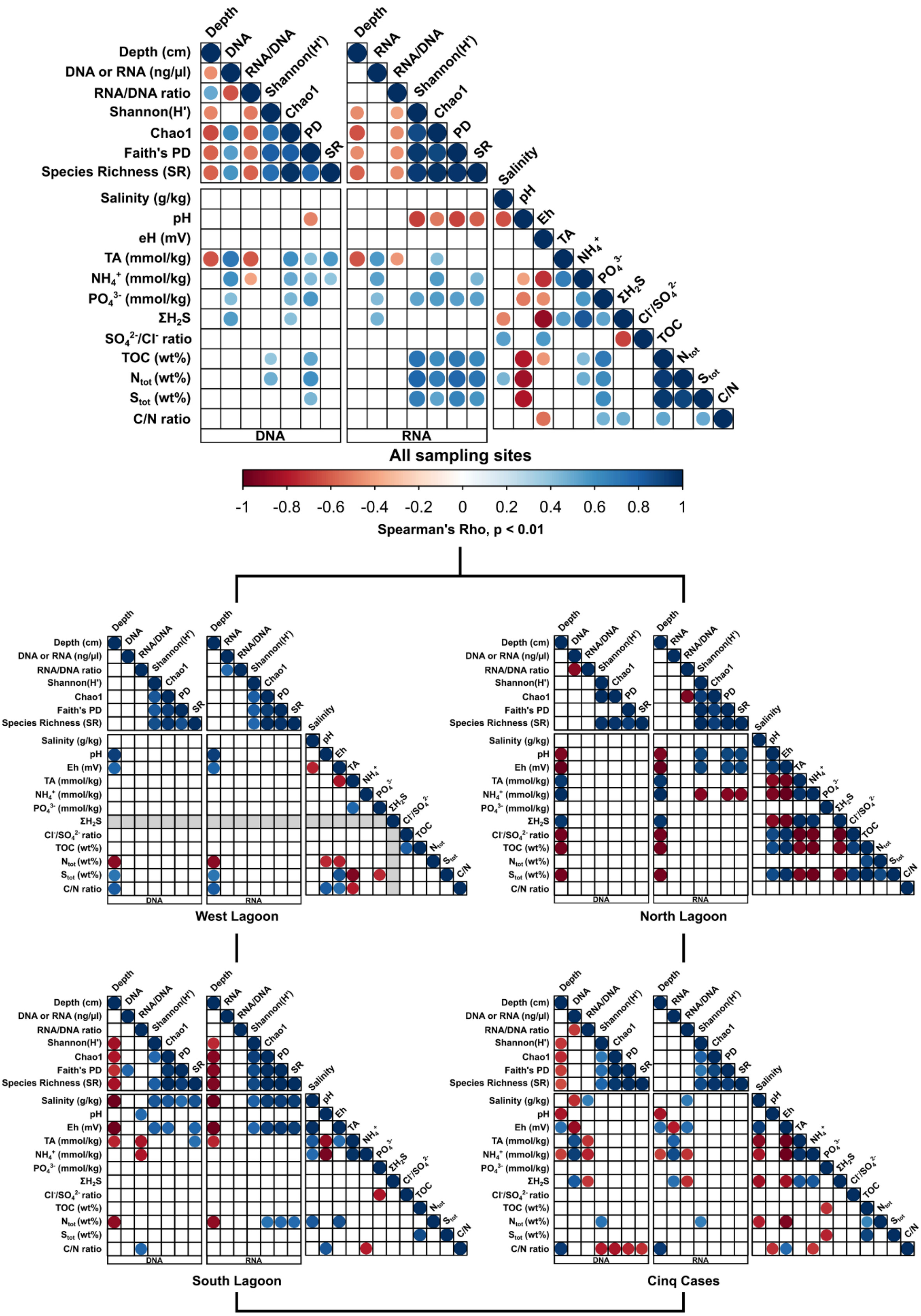

Supplementary Figure S1. Spearman's correlations of mean diversity and richness indices per depth with sediment porewater data. The data were correlated together and by sampling site to identify general trends. Water samples were excluded from the analysis, as they represent a separate environment. Significant correlations were plotted if $p<0.01$. The statistical results including p- and Rho-values can be found in Supplementary Table S2.
