# Supplementary material for: DNA- and RNA-based bacterial communities and geochemical zonation under changing sediment porewater dynamics on the Aldabra Atoll
Source: Sci Rep. 2022 Mar 11;12:4257. doi: 10.1038/s41598-022-07980-0 (PMC8917147; doi:10.1038/s41598-022-07980-0)
Supplement: Supplementary file 4 — Supplementary Figure 3. [file 41598_2022_7980_MOESM4_ESM.pdf]

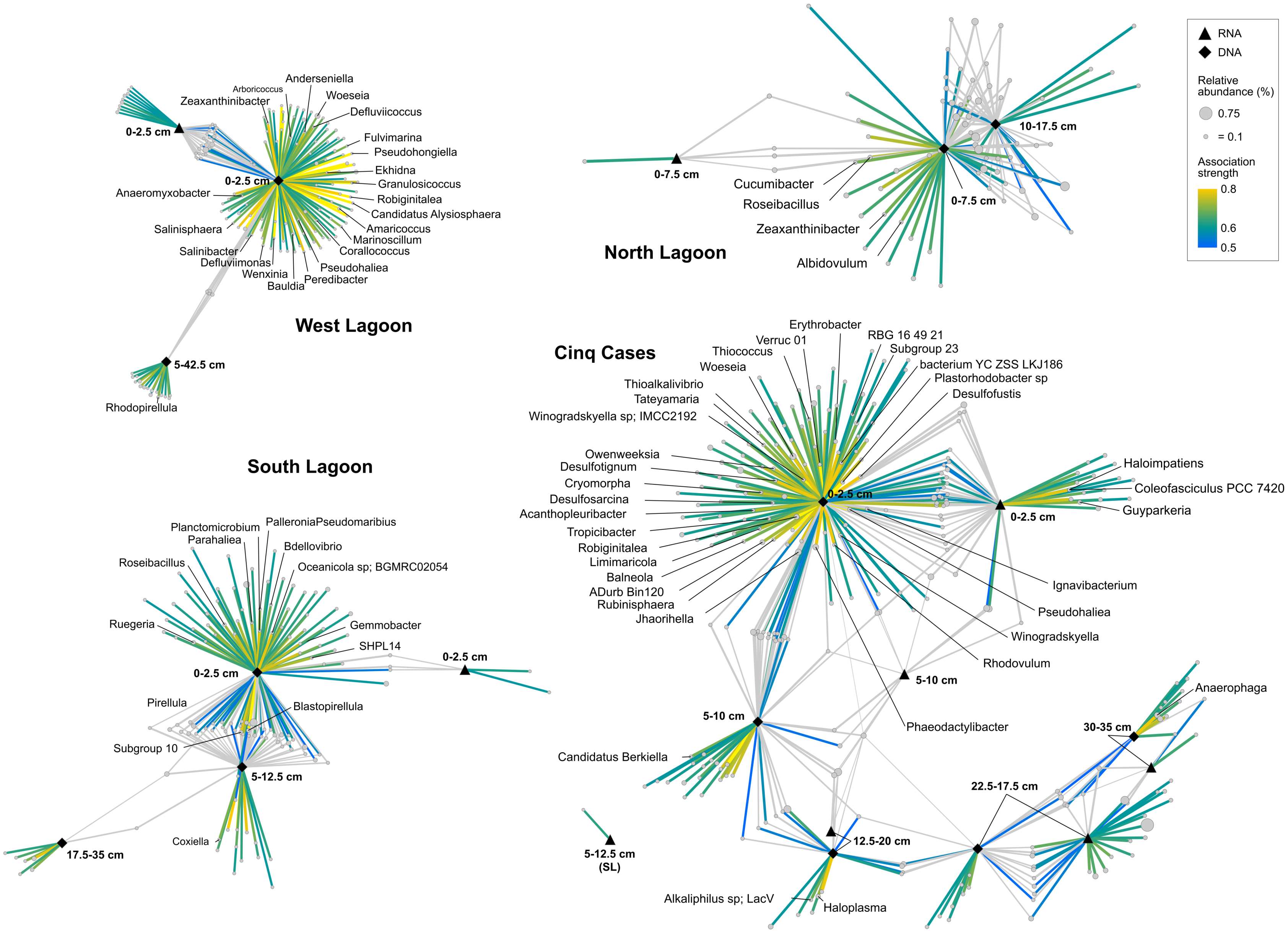

Supplementary Figure S3. Association networks of the rare taxa in the total and active bacterial community of each geochemical zone. The abundance cut-off was set to < 2 % for all sites except Cinq Cases where <5 % was used. Named genera with an association strength above 0.7 are indicated. Edge colour and width are scaled according to the association strength of each node to the target sediment zone.
