# Supplementary material for: DNA- and RNA-based bacterial communities and geochemical zonation under changing sediment porewater dynamics on the Aldabra Atoll
Source: Sci Rep. 2022 Mar 11;12:4257. doi: 10.1038/s41598-022-07980-0 (PMC8917147; doi:10.1038/s41598-022-07980-0)
Supplement: Supplementary file 5 — Supplementary Figure 4. [file 41598_2022_7980_MOESM5_ESM.pdf]

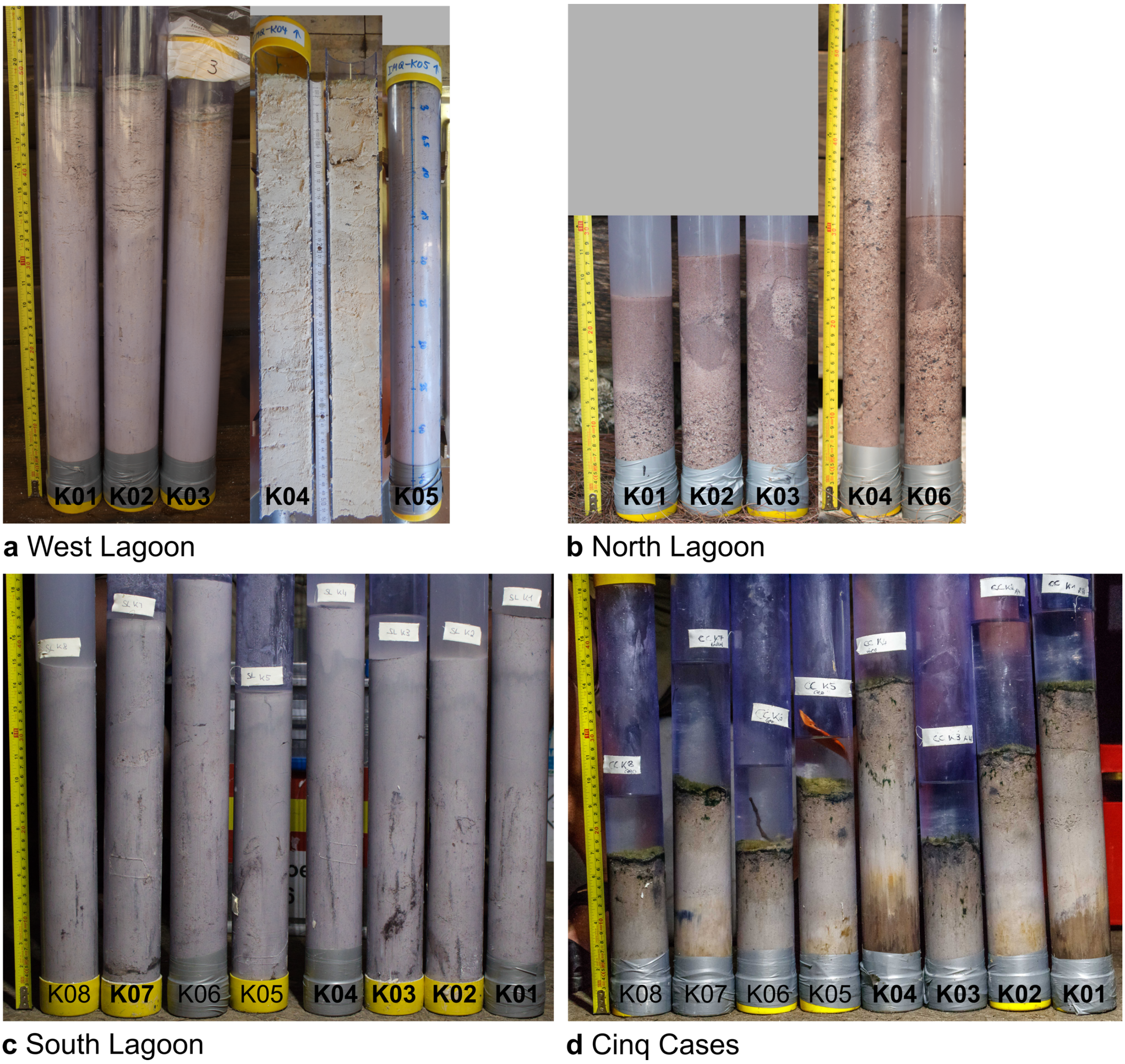

Supplementary Figure S4. Original photographs of all sediment cores from the different sampling sites. Cores K01-03 of each site were used for bacterial community analysis. Cores K04 were used for bulk chemistry measurements at all sampling sites. Core K04 was also used for porewater chemistry measurement at Cinq Cases. For the remaining site porewaters were measured in separate cores, namely K04 in the West Lagoon, K06 in the North Lagoon and K07 in the South Lagoon. All used cores are highlighted in bold.
