# Supplementary material for: DNA- and RNA-based bacterial communities and geochemical zonation under changing sediment porewater dynamics on the Aldabra Atoll
Source: Sci Rep. 2022 Mar 11;12:4257. doi: 10.1038/s41598-022-07980-0 (PMC8917147; doi:10.1038/s41598-022-07980-0)
Supplement: Supplementary file 3 — Supplementary Figure 2. [file 41598_2022_7980_MOESM3_ESM.pdf]

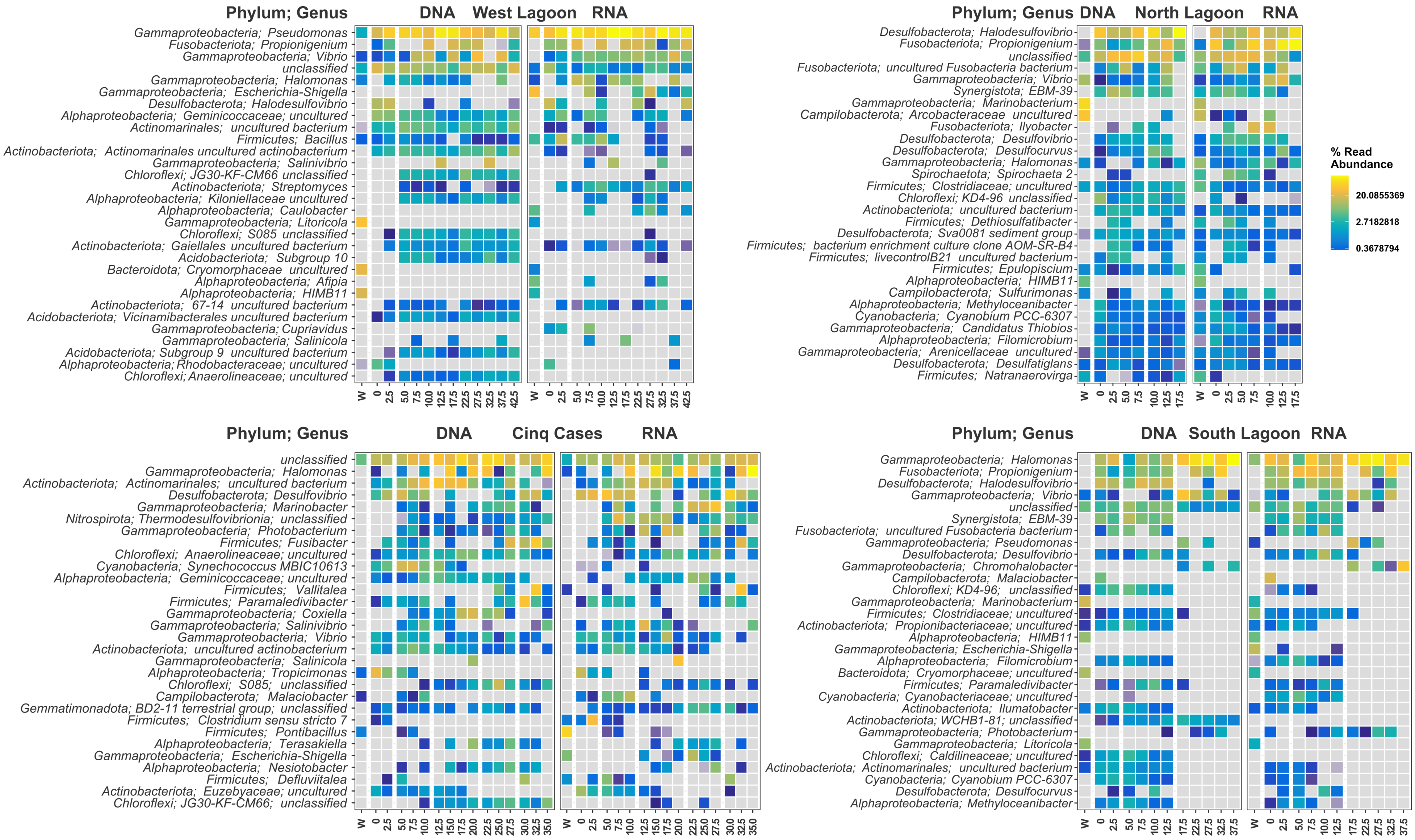

Supplementary Figure S2. Heatmap of the 30 most abundant genera in the total (DNA) and active (RNA) bacterial community. Replicates were normalized, transformed into relative abundances, and averaged by depth. Gaps indicate the geochemical zonation.
